# HEV Infection in Beta-Thalassemia Patients

**DOI:** 10.3390/pathogens13121058

**Published:** 2024-12-01

**Authors:** Despoina Papageorgiou, Anne-Lise de Lastic, Olga Tsachouridou, Simeon Metallidis, Karolina Akinosoglou

**Affiliations:** 1 Medical School, University of Patras, Rio, 26504 Patras, Greece; dspn.pap96@gmail.com; 2 Laboratory of Immunohematology, Medical School, University of Patras, Rio, 26504 Patras, Greece; delastic@gmail.com; 3 Department of Internal Medicine and Infectious Diseases, AHEPA Hospital, Aristotle University of Thessaloniki, 54636 Thessaloniki, Greece; olgatsachouridou.iasis@gmail.com (O.T.); symeonam@auth.gr (S.M.); 4 Department of Internal Medicine and Infectious Diseases, University General Hospital of Patras, Rio, 26504 Patras, Greece

**Keywords:** HEV, hepatitis E, thalassemia, beta-thalassemia, prevalence, risk factors, clinical impact, prevention

## Abstract

Thalassemia is an inherited hematological disorder characterized by a decrease in the synthesis of or absence of one or more globin chains. Hepatitis E virus (HEV) is a major cause of acute viral hepatitis, constituting a major global health burden and emerging as a critical public health concern. HEV infection is mainly transmitted via the fecal–oral route; however, parenteral transmission through blood components has been reported in both developing and developed countries. Although HEV infection is typically self-limiting, immunocompromised individuals, patients with chronic liver disease, and thalassemic patients are at a heightened risk of contracting the infection and may develop chronic hepatitis and life-threatening complications that require treatment. The reported prevalence rates of HEV in thalassemia patients vary significantly by country. Age, gender, residential area, and the cumulative amount of blood transfusions received have been identified as associated risk factors for HEV infection. In order to enhance blood safety and ensure the protection of vulnerable patient populations, such as thalassemia patients, several countries have introduced universal or targeted HEV screening policies in blood donations. Other preventive measures include vigilant monitoring of thalassemic patients and screening for anti-HEV antibodies. The aim of this review is to explore the prevalence, risk factors, clinical impact and management of HEV infection in patients with thalassemia.

## 1. Introduction

Thalassemia is an inherited hematological disorder characterized by a decrease in the synthesis of or absence of one or more globin chains. Beta-thalassemia is a result of one or more mutations in the beta-globin gene and can be further divided into three main types: thalassemia major, intermedia, and minor/carrier state [1]. In thalassemia major, lifelong transfusions are required for survival (transfusion-dependent thalassemia—TDT), whereas in thalassemia intermedia the patients are not routinely transfused but may require occasional or even frequent transfusions in certain clinical settings and for defined periods of time (non-transfusion-dependent—NTDT). Beta-thalassemia is highly prevalent in the Mediterranean, Middle East, and Central and Southeast Asia regions; however, due to population shifts, it is becoming more common worldwide, making the management and care of thalassemic patients an increasing concern for the healthcare systems globally [2]. Clinical manifestation varies from typically asymptomatic in carrier states to severe anemia in thalassemia major, which requires regular management with blood transfusions [3]. A typical challenge in the treatment of thalassemic patients is iron overload, resulting from blood transfusion therapy and increased gastrointestinal absorption [4]. This condition necessitates vigilant monitoring and the implementation of iron chelation therapy in order to prevent damage to target organs [1,5]. For the purposes of this review, our focus is on transfusion-receiving thalassemia patients (mainly TDT and some cases of NTDT), whose regular and heavy transfusion load puts them at a higher exposure-related danger of infection by blood-borne pathogens and whose multiple co-morbidities make them more vulnerable to infection complications.

Hepatitis E virus (HEV) is a major cause of acute viral hepatitis, with an estimated 20 million infections worldwide, leading to approximately 3.3 million symptomatic cases of hepatitis E [6]. A high prevalence of HEV infection has been reported in Africa, Central and Southeast Asia, and Mexico [7]. In France, the seroprevalence of HEV (anti-HEV immunoglobulin G (IgG) and IgM) ranged from 21.9% to 71.3%, depending on geographic location, with an average of 39.1% [8]. Hepatitis E exhibits a global distribution characterized by two distinct epidemiological patterns. In developing countries, an endemic disease pattern is observed, with HEV infection primarily caused by genotypes 1 and 2. Transmission occurs via the fecal–oral route due to contaminated drinking water and inadequate sanitary conditions. These genotypes are more prevalent in tropical areas, such as Asia, parts of Africa, and Central America. Conversely, in Europe, Australia, and North and South America, HEV genotypes 3 (in the most part) and 4 are responsible for the emergence of sporadic cases of the disease, which are typically transmitted zoonotically, especially through the consumption of undercooked meat from infected animals [9,10,11]. Other transmission routes of HEV have been described, including direct contact with infected animals, vertical transmission, and notably parenteral transmission following blood and blood product transfusions [9,12]. Individuals with lower levels of education and access to freshwater amenities may be at greater risk of exposure and infection compared to those with higher educational attainment and better living conditions, mainly in the areas where genotypes 1 and 2 are prevalent. Although in most cases HEV infection remains asymptomatic or mildly symptomatic, with the disease following a self-limiting course, acute icteric hepatitis is observed in 5%–30% of infected patients [13,14].

Of importance, immunocompromised individuals have been identified as high-risk patients for developing chronic HEV infection that may result in liver damage and cirrhosis [15,16].

Infectious diseases and their complications are currently becoming the leading cause of mortality in Western countries among transfusion-dependent thalassemia patients [17]. A wide spectrum of immune deficits has been observed in beta-thalassemia patients, which may contribute to poor outcomes following infection [18]. In addition, iron overload may exacerbate the progression and severity of viral hepatitis, leading to increased morbidity and mortality in these patients [19]. Therefore, there is a growing concern about transfusion-transmitted HEV, which underscores the importance of investigating HEV infection in thalassemia patients [12]. Moreover, due to the shift in populations due to immigration in recent decades, large numbers of thalassemic patients currently reside in all areas where different genotypes are prevalent.

The main objective of this article is to review and discuss the existing literature on HEV infection in TDT and NTDT thalassemia patients. Specifically, this review aims to determine the reported prevalence of HEV infection in thalassemia patients, to identify the associated risk factors for HEV infection in this population, and to assess the clinical impact and review the management and treatment strategies for HEV infection in thalassemic patients.

## 2. Methods

**Figure A1:**
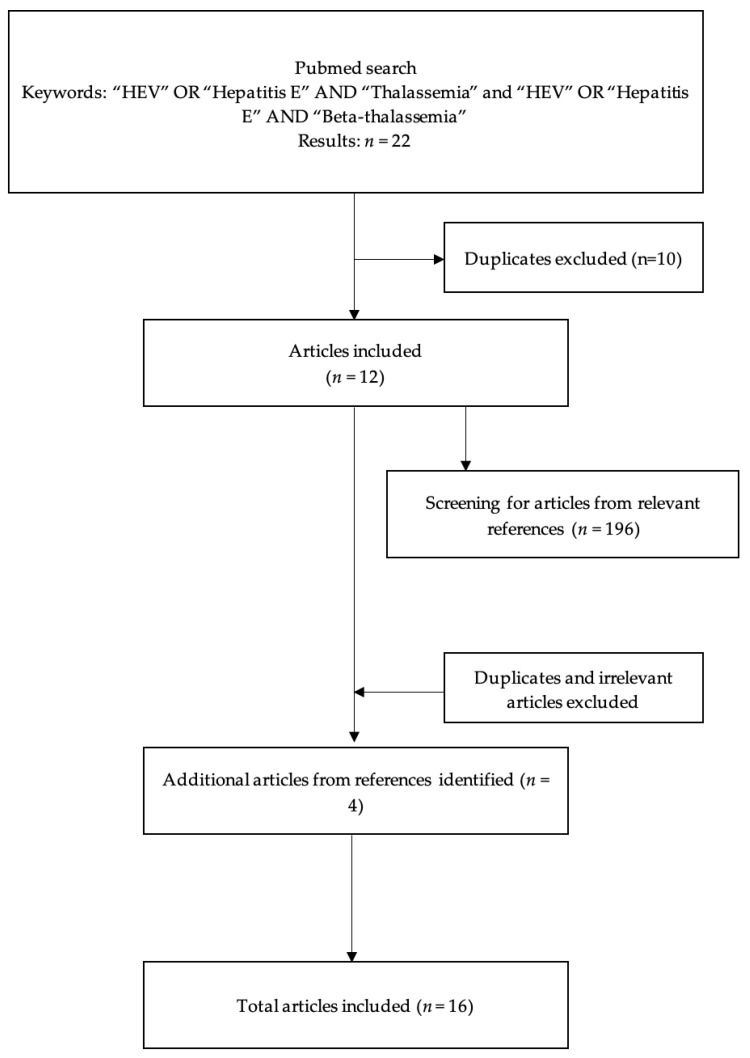
Flowchart of the review methodology. After an initial keyword-based search, 12 articles were selected and then their references were screened. Any additional papers relevant to the review were included in the final study pool, bringing the total number of studies on HEV infection on thalassemia patients to 16.

We performed a literature search in the PubMed database from 1996 until June 2024 by using the terms “HEV” OR “Hepatitis E” AND “beta-thalassemia”, “HEV” OR “Hepatitis E” AND “thalassemia”. Only English-language papers were reviewed. Relevant publications were identified by title and abstract screening by two independent reviewers. Respective articles were also hand-searched in order to identify relevant references. Duplicates, as well as irrelevant articles, were excluded. The results were discussed, and disagreements resolved. Appendix A contains a flowchart of our methodology and the included results (Figure A1).

## 3. Results

Table 1 summarizes the characteristics of the studies included in this review, providing information including the author, year, study type, patient population, and the main findings of each study. The populations studied primarily included patients with transfusion-dependent thalassemia, with various sample sizes across studies, ranging from 28 to 150 patients with thalassemia. The studies were conducted in diverse geographical locations, mainly comprising regions where HEV is endemic, such as Iran (Farshadpour et al., Bineshian et al., Dalvand et al., Golshan et al., Elizee et al., Jahromi et al.), Egypt (Abdeimawala et al.), India (Arshad et al.), Saudi Arabia (Al Fawatz), but also developed countries including Greece (Dalekos et al., Psichogiou et al., Klozinakis et al.) and Brazil (Slavov et al.). Prevalence ranged from 0 to 27.15%, while no cases of viremia were recorded. Seroprevalence was higher among thalassemia patients compared to the general population (Farshadpour et al., Slavov et al., Al Fawaz et al.) but not in all settings (Dalvand et al., Golshan et al.). The number of transfusions was identified as a potential risk factor of seropositivity in a number of studies (Farshadpour et al., Abdewmawla) but not in all cases (Bineshian et al., Slavov et al., Psichogiou et al.). Other associated factors included older age, residency in rural areas, and elevated liver enzymes (Abdelmawla et al., Jahromi et al., Psichogiou et al.).

## 4. Discussion

In this report, we aimed to explore the prevalence of HEV infection in thalassemia patients. First, we found that the prevalence of HEV in thalassemia patients varied significantly by country.

The highest prevalence rate among thalassemia patients was observed in Egypt, with an overall seroprevalence rate of 27.15% [20], in line with respective studies among HCV- or HBV-seropositive blood donors, revealing an HEV prevalence of 28.76% [33]. This observation, in combination with the fact that among healthy blood donors, 3 out of 760 samples (0.45%) were positive for anti-HEV IgM—two of which positive for HEV RNA—suggests that blood transfusion remains a potential route of HEV transmission in Egypt [34,35].

In Iran, HEV seroprevalence rates differed substantially by region. Recent studies from southern territories reported a 10–16.67% prevalence of HEV among thalassemia patients, significantly higher than the general population [21,25]. Conversely, previous studies from Iran have revealed lower seroprevalence rates in thalassemic patients. A study on 120 HCV-positive thalassemia patients showed that 1.67% were positive for anti-HEV antibodies [27]. A study in northeast Iran found that 2.6% of beta-thalassemia major patients had HEV infection compared to 8.6% of healthy controls [24]. Another study reported that 4.5% of thalassemic patients tested positive for anti-HEV antibodies [22]. In an older study of 64 HCV-positive thalassemic patients, only 1.6% tested positive for anti-HEV antibodies [26].

HEV prevalence rates of 13% and 10.7% were documented in thalassemic patients in Brazil and Saudi Arabia, respectively [31,32], with a higher HEV seropositivity rate compared to that of the general population (3.0%) [36]. Similar to Egypt, HEV seroprevalence in Brazilian blood donors ranges from 0.9% to 18.7% in different geographic regions [37,38,39,40,41].

Relative rates from studies conducted in Greece indicated a low prevalence of HEV, ranging from 0.0% to 2.4% among thalassemia patients [29,30]. Higher rates of HEV seroprevalence were found among healthy blood donors and people living with HIV. In a study comprising 265 healthy individuals, 25 samples (9.43%) tested positive for anti-HEV IgG [42]. In a recent study comprising HIV positive patients, the prevalence of HEV IgG and IgM antibodies was 16.5% and 8.6%, respectively, while active viral replication (HEV RNA) was present in 2.3% of patients [43]. A study conducted in Greece investigating the prevalence of HEV among high-risk individuals, including 125 thalassemia patients, HIV-positive individuals, and intravenous drug users (IVDUs), found an anti-HEV IgG prevalence rate of 2.4% in the thalassemia group. The overall prevalence rate was 3.5%, compared to 2.2% in healthy controls, a difference that was not statistically significant [29]. Other authors reported that among 40 patients with hereditary hemoglobinopathies, such as beta-thalassemia and sickle cell disease, no one tested positive for anti-HEV IgG and IgM [28]. A contemporary study encompassing 96 transfusion-dependent thalassemia patients demonstrated that all samples tested negative for both HEV RNA and anti-HEV IgG, suggesting a low incidence of HEV infection among thalassemic patients in Greece [30].

When assessing the prevalence of HEV in the general population, it is crucial to consider the variability in infection rates and the diverse risk factors across different global regions. Additionally, the heterogeneity of study protocols used to determine seroprevalence should be acknowledged. These protocols range from analyses of healthy blood donor banks and individuals with acute hepatitis to broader studies of the general population. Variations in HEV seroprevalence have been observed in other patient populations as well. HEV seroprevalence in the general Iranian population ranges from 1.1% to 14.2%. The respective rates are 4.5–14.3% in healthy blood donors, 6.3%–28.3% among hemodialysis patients, 1.6–11.3% among patients with other hepatitis virus coinfection, and 10–16.4% in patients with human immunodeficiency virus. These variations reflect differences in public health status, risk factors, and routes of transmission in different regions and patient groups [44].

A number of studies have explored potential risk factors for HEV infection. Increased age, elevated liver enzymes, as well as living settings have been identified [20,25,29]. A study conducted in Egypt found a significant difference in HEV prevalence between rural and urban residential areas, with a higher prevalence of HEV in rural regions [20], suggesting that socioeconomic status and hygiene measures are predominant factors of disease. Although HEV is mainly transmitted by the fecal–oral route, parenteral transmission through blood transfusions has been reported in both developing and developed countries, since the incidence of viremic donors is surprisingly high [45,46]. This comes in line with the finding that several authors reported a significant association between the amount of blood transfusions received and anti-HEV seropositivity [20,21]. However, this was not a universal finding [23,29].

HEV infection usually results in self-limited disease; however, immunocompromised individuals, patients with pre-existing chronic liver disease, and patients with transfusion-dependent thalassemia are at an increased risk of developing chronic infection and life-threatening complications [20,47]. Importantly, several authors have noted that thalassemia patients are at an increased risk of developing chronic liver disease, including chronic HCV infection [20,26]. Progression to severe illness can lead to complications such as acute liver failure, cirrhosis, and neurological dysfunction, and is also associated with increased morbidity and mortality rates [25,26,30]. However, only a limited number of case reports have documented the clinical impact of HEV infection in thalassemia patients. In a reported case, a newly diagnosed thalassemic patient developed non-focal abdominal discomfort, decreased appetite, worsening jaundice, and deteriorating liver function tests. The serological studies were consistent with acute HEV infection [48]. Another case report described a rare coexistence of severe intravascular hemolysis in a patient with hepatitis E and beta thalassemia trait [49]. With regard to laboratory abnormalities, a study conducted in Egypt found that 84.2% and 68.4% of patients with HEV seropositivity showed elevated ALT and AST levels, respectively. However, the study demonstrated that liver enzymes were higher among patients with non-HEV antibodies compared to those with HEV antibodies. Among anti-HEV IgM-positive patients, 100% had elevated ALT and 75% had elevated AST levels, although there was no significant association between liver enzymes and anti-HEV IgM seropositivity [20]. Results from another study indicated that the serum levels of ALT and AST were significantly higher in the anti-HEV IgG- and IgM-positive patients than those in the anti-HEV-negative patients [25]. In contrast, serum levels of liver enzymes were not statistically associated with anti-HEV seroprevalence in another study from Iran [21].

Several studies demonstrate that thalassemic patients display an altered immunological profile both in the innate and the adaptive branch of the immune system. There seems to be a complement defect, disrupted chemotaxis and phagocytosis, decreased NK and T cell activation, and immunosenescence, leading to a profile of increased susceptibility to infections (both bacterial and viral). Constant alloantigenic challenge due to transfusions, iron dysregulation, zinc deficiency, and chelation-related immunomodulation have been implicated as culprits. Moreover, in the event of an infection, multiple co-morbidities beginning with hepatic iron overload set the stage for a complicated disease progression. Due to the paucity of data on HEV infection in this population, we do not have good-quality data regarding its clinical course and therefore have to rely on case reports and limited epidemiologic observations.

Management and treatment of HEV infection in beta-thalassemia patients does not seem to differ among other patient populations, including immunocompromised patients and those with chronic liver disease, and depends on disease severity. Since HEV infection could lead to a severe clinical form in individuals with pre-existing liver disease, testing for anti-HEV IgM antibodies and HEV viral load assessment are recommended in cases of acute hepatitis or unexplained flares of chronic liver disease [50]. In severe cases, treatment guidelines include ribavirin and IFN (although ribavirin can cause hemolysis and IFN cytopenias, thus complicating their use); however, further investigation is warranted [50]. In a single case report involving a beta-thalassemia patient with acute HEV infection, the authors noted that there was no requirement for significant long-term alternations in transfusion or chelation strategy due to the self-limiting and non-severe disease course in their specific case [48]. To this end, the necessity of preventive measures in order to enhance blood safety and ensure the protection of vulnerable patient populations is highlighted. In this regard, several countries have adopted a universal screening policy for HEV detection in blood donations, while other countries have introduced a selective HEV screening strategy examining blood components that are intended for high-risk patient populations [12,51]. In Europe, England, Ireland, the Netherlands, Switzerland, France, Austria, and Germany have started to include HEV in routine testing of blood products and the EASL issued a statement in 2019 advising routine HEV screening [10,52]. Australia conducted a large-scale epidemiologic survey and in 2017 concluded that, due to very low local prevalence, no routine HEV testing would be implemented in blood donations [53]. Despite this, clinicians should maintain a heightened clinical suspicion for HEV infection among patients with thalassemia. Several authors recommend monitoring thalassemia patients by assessing liver function tests to identify subclinical HEV infection and advocate for screening these patients for anti-HEV antibodies [24,27].

Several limitations were observed in the reviewed studies. A common issue across the studies was the relatively small sample sizes, which may impact the generalizability of the results. Another notable limitation is that some studies did not evaluate HEV RNA or anti-HEV IgM, instead only measuring anti-HEV IgG antibodies. The omission of these markers of acute infection may underestimate the overall HEV prevalence. One limitation of the current study is that it is restricted to only a few geographical locations. As the epidemiology of HEV varies significantly across different regions, future research should address this geographic restriction and should aim to overcome the sample size limitations. Additionally, further research is required to investigate the clinical impact and management of HEV infection in patients with thalassemia.

## 5. Conclusions

HEV seroprevalence in patients with thalassemia varies significantly by country. Older age, gender, the amount of blood transfusions, and residential area have been reported as associated risk factors for HEV infection. HEV is primarily transmitted via the fecal–oral route; however, parenteral transmission has been reported in both developed and developing countries. HEV infection is usually self-limiting, although immunocompromised individuals, including patients with thalassemia, may develop a more severe disease course and associated complications. In severe cases, treatment with ribavirin and IFN is recommended. Prevention measures include universal or selective screening of blood donations for anti-HEV antibodies and HEV RNA. In thalassemia patients, monitoring for liver enzyme elevations and screening for anti-HEV antibodies is also suggested.

## Figures and Tables

**Table 1:** This table includes details on study design, author, year, sample size, population characteristics, and key findings of the reviewed studies on HEV infection in thalassemia patients.

Region	Author	Study Type	Population	Key Findings
EGYPT	Abdelmawla et al. 2019 [20]	Cross-sectional study	140 patients with β-thalassemia Southern Iran	The seroprevalence of HEV antibodies was **27.15%** (38 out of 140 patients 34 patients (24.29%) were positive for anti-HEV IgG and 4 patients (2.86%) were positive for anti-HEV IgM
IRAN	Farshadpour et al. 2022 [21]	ND	120 β-thalassemia patients 124 healthy controls	**16.67%** of thalassemia patients were positive for anti-HEV IgG compared to 12.1**%** of the controls, suggesting high seroprevalence among thalassemia patients All patients were negative for anti-HEV IgM and HEV viremia
	Bineshian et al. 2020 [22]	Observational cross-sectional study	110 thalassemia patients	5 out of 110 (**4.5%**) patients were positive for anti-HEV ab (IgG or IgM) No sample was positive for HEV-RNA
	Dalvand et al. 2019 [23]	Cross-sectional study	120 anti-HCV positive thalassemia patients	2 out of 120 (**1.67%**) were positive for anti-HEV antibodies (IgG and IgM) None of the 120 samples were positive for HEV-RNA
	Golshan et al. 2017 [24]	Cross-sectional study	150 β-thalassemia major patients and 150 healthy controls	4 thalassemia patients (**2.6%**) had HEV infection compared to 13 healthy controls (8.6%)
	Jahromi et al. 2016 [25]	Cross-sectional study	110 thalassemic patients Southern Iran	11 out of 110 (**10%**) patients were HEV IgG-positive and 2 out of 110 (**1.8%**) were HEV IgM-positive Higher HEV seropositivity rate was found among patients aged 11–20 years
	Elizee et al. 2013 [26]	Cross-sectional study	64 thalassemic patients and 155 patients with hemophilia (with concurrent HCV infection)	HEV seropositivity was low, only 1 thalassemia patient (**1.6%**) and 5 hemophilia patients (3.2%) tested positive for anti-HEV abs
INDIA	Irshad et al. 2002 [27]	ND	50 thalassemic patients	Recent HEV infection was present in 5 patients (**10%**)
GREECE	Dalekos et al. 1998 [28]	Cross-sectional study	40 patients with hemolytic hemoglobinopathies (β-thalassemia and sickle cell disease)	No patient tested positive for anti-HEV IgG or IgM
	Psichogiou et al. 1996 [29]	Observational, cross-sectional study	125 thalassemia patients (the study included other high risk patient groups total number of patients was 1155)	Prevalence of anti-HEV IgG among thalassemia patient group was **2.4%**, with an overall prevalence in high-risk individuals of 3.5%. Not statistically significant compared to 2.2% in healthy controls
	Klonizakis et al. 2017 [30]	Retrospective observational study	96 transfusion-dependent thalassemia patients	All samples were negative for HEV RNA Anti-HEV IgG antibodies were negative in all measured samples
SAUDI ARABIA	Al-Fawaz et al. 1996 [31]	Observational study	28 patients with β thalassemia major and 50 with sickle cell disease	In thalassemia group, HEVab was found in **10.7%** of patients compared to 2.8% in healthy control group (not statistically significant), while HCVab was found in 57.1% versus 1.4% in healthy controls
BRAZIL	Slavov et al. 2019 [32]	Cross-sectional observational study	40 thalassemia patients and 52 with sickle cell disease	The anti-HEV IgG seroprevalence was significantly higher in thalassemia patients compared to the rate in sickle cell disease patients (**20%** versus 7.7% respectively) No HEV-RNA was detected in either study group

Abbreviations: ND: no data available; HEV: hepatitis E virus; HCV: hepatitis C virus; HBV: hepatitis B virus; HAV: hepatitis A virus; IgG: immunoglobulin G; IgM: immunoglobulin M; ab: antibodies; HIV: human immunodeficiency virus.

## References

[1] Ali S., Mumtaz S., Shakir H.A., Khan M., Tahir H.M., Mumtaz S., Mughal T.A., Hassan A., Kazmi S.A.R., Sadia (2021). Current Status of Beta-Thalassemia and Its Treatment Strategies. Mol. Genet. Genom. Med..

[2] Motta I., Bou-Fakhredin R., Taher A.T., Cappellini M.D. (2020). Beta Thalassemia: New Therapeutic Options Beyond Transfusion and Iron Chelation. Drugs.

[3] Needs T., Gonzalez-Mosquera L.F., Lynch D.T. (2023). Beta Thalassemia.

[4] Origa R. (2017). β-Thalassemia. Genet. Med..

[5] Asadov C., Alimirzoeva Z., Mammadova T., Aliyeva G., Gafarova S., Mammadov J. (2018). β-Thalassemia Intermedia: A Comprehensive Overview and Novel Approaches. Int. J. Hematol..

[6] Hepatitis E. https://www.who.int/news-room/fact-sheets/detail/hepatitis-e.

[7] Li P., Liu J., Li Y., Su J., Ma Z., Bramer W.M., Cao W., de Man R.A., Peppelenbosch M.P., Pan Q. (2020). The Global Epidemiology of Hepatitis E Virus Infection: A Systematic Review and Meta-Analysis. Liver Int..

[8] Clemente-Casares P., Ramos-Romero C., Ramirez-Gonzalez E., Mas A. (2016). Hepatitis E Virus in Industrialized Countries: The Silent Threat. BioMed Res. Int..

[9] Raji Y.E., Toung O.P., Taib N.M., Sekawi Z.B. (2022). Hepatitis E Virus: An Emerging Enigmatic and Underestimated Pathogen. Saudi J. Biol. Sci..

[10] Wolski A., Pischke S., Ozga A.-K., Addo M.M., Horvatits T. (2023). Higher Risk of HEV Transmission and Exposure among Blood Donors in Europe and Asia in Comparison to North America: A Meta-Analysis. Pathogens.

[11] Songtanin B., Molehin A.J., Brittan K., Manatsathit W., Nugent K. (2023). Hepatitis E Virus Infections: Epidemiology, Genetic Diversity, and Clinical Considerations. Viruses.

[12] Cheung C.K.M., Law M.F., Wong S.H., Law A.W.H. (2022). Transfusion-Transmitted Hepatitis E: What We Know so Far?. World J. Gastroenterol..

[13] Lhomme S., Marion O., Abravanel F., Izopet J., Kamar N. (2020). Clinical Manifestations, Pathogenesis and Treatment of Hepatitis E Virus Infections. J. Clin. Med..

[14] Aslan A.T., Balaban H.Y. (2020). Hepatitis E Virus: Epidemiology, Diagnosis, Clinical Manifestations, and Treatment. World J. Gastroenterol..

[15] Thakur V., Ratho R.K., Kumar S., Saxena S.K., Bora I., Thakur P. (2020). Viral Hepatitis E and Chronicity: A Growing Public Health Concern. Front. Microbiol..

[16] Alexandrova R., Tsachev I., Kirov P., Abudalleh A., Hristov H., Zhivkova T., Dyakova L., Baymakova M. (2024). Hepatitis E Virus (HEV) Infection Among Immunocompromised Individuals: A Brief Narrative Review. Infect. Drug Resist..

[17] Farmakis D., Porter J., Taher A., Cappellini M.D., Angastiniotis M., Eleftheriou A., Alassaf A., Angastiniotis M., Angelucci E., Aydinok Y. (2022). 2021 Thalassaemia International Federation Guidelines for the Management of Transfusion-Dependent Thalassemia. HemaSphere.

[18] Gluba-Brzózka A., Franczyk B., Rysz-Górzyńska M., Rokicki R., Koziarska-Rościszewska M., Rysz J. (2021). Pathomechanisms of Immunological Disturbances in β-Thalassemia. Int. J. Mol. Sci..

[19] Mancinelli R., Rosa L., Cutone A., Lepanto M.S., Franchitto A., Onori P., Gaudio E., Valenti P. (2020). Viral Hepatitis and Iron Dysregulation: Molecular Pathways and the Role of Lactoferrin. Molecules.

[20] Abdelmawla D., Moemen D., Darwish A., Mowafy W. (2019). Hepatitis E Virus Prevalence in Egyptian Children with Transfusion-Dependent Thalassemia. Braz. J. Infect. Dis..

[21] Farshadpour F., Taherkhani R., Shaeri M. (2022). Prevalence and Risk Factors of Hepatitis E Virus Infection among Patients with β-Thalassemia Major in South of Iran. J. Immunoass. Immunochem..

[22] Bineshian F., Dalvand N., Hosseini S.M., Sharifi Z. (2020). Hepatitis E Virus Infection Among Iranian Patients with Beta-Thalassemia. Middle East J. Rehabil. Health Stud..

[23] Dalvand N., Dalvand A., Sharifi Z., Hosseini S.M. (2019). Prevalence of Hepatitis E Virus in Thalassemia Patients with Hepatitis C in Tehran, Iran. Iran. J. Microbiol..

[24] Golshan A., Abrishami F. (2017). Comparison Between the Frequency of Hepatitis E Virus Among Major Thalassemia Patients With Control Group in Mashhad, North East of Iran. Int. J. Infect..

[25] Jahromi A.S., Ahmadi-vasmehjani A., Zabetian H., Hakimelahi H., Yusefi A., Sanie M.S., Jahromi S.T., Ghanei M., Sapidkar A., Erfanian S. (2016). Sero-Epidemiological Study of Hepatitis E Virus among Thalassemia as High Risk Patients: A Cross-Sectional Survey in Jahrom, Southern, Iran. Glob. J. Health Sci..

[26] Elizee P.K., Alavian S.M., Miri S.M., Behnava B., Alavian S.H., Keshvari M., Fesharaki M.G., Salimi S., Mehrnoush L., Shafiei M. (2013). The Seroprevalence of Entrically Transmitted Viral Hepatitis in HCV Infected Thalassemia and Hemophilia Patients in Iran. Jundishapur J. Microbiol..

[27] Irshad M., Peter S. (2002). Spectrum of Viral Hepatitis in Thalassemic Children Receiving Multiple Blood Transfusions. Indian J. Gastroenterol. Off. J. Indian Soc. Gastroenterol..

[28] Dalekos G.N., Zervou E., Elisaf M., Germanos N., Galanakis E., Bourantas K., Siamopoulos K.C., Tsianos E.V. (1998). Antibodies to Hepatitis E Virus among Several Populations in Greece: Increased Prevalence in an Hemodialysis Unit. Transfusion.

[29] Psichogiou M., Tzala E., Boletis J., Zakopoulou N., Loutradi A., Maliori M., Kourea-Kremastinou J., Stratigos J., Hatzakis A. (1996). Hepatitis E Virus Infection in Individuals at High Risk of Transmission of Non-A, Non-B Hepatitis and Sexually Transmitted Diseases. Scand. J. Infect. Dis..

[30] Klonizakis P., Gioula G., Exindari M., Apostolou C., Kotsiafti A., Vlachaki E. (2017). Hepatitis E in Transfusion-Dependent Thalassaemia Patients, in Greece: A Single Centre Experience. Vox Sang..

[31] Al-Fawaz I., Al-Rasheed S., Al-Mugeiren M., Al-Salloum A., Al-Sohaibani M., Ramia S. (1996). Hepatitis E Virus Infection in Patients from Saudi Arabia with Sickle Cell Anaemia and β-Thalassemia Major: Possible Transmission by Blood Transfusion. J. Viral Hepat..

[32] Slavov S.N., Maçonetto J.D.M., Martinez E.Z., Silva-Pinto A.C., Covas D.T., Eis-Hübinger A.M., Kashima S. (2019). Prevalence of Hepatitis E Virus Infection in Multiple Transfused Brazilian Patients with Thalassemia and Sickle Cell Disease. J. Med. Virol..

[33] Ahmed A.M.A., Temerk H.A., Galal H.R., Bazeed S.E.S., Sultan S. (2020). The Seropervelance of Infectious Hepatitis Viruses (HBV, HCV and HEV) among Blood Donors and Their Correlation to Risk Factors in Qena Governorate, Upper Egypt. VirusDisease.

[34] Martino B.D., Profio F.D., Palombieri A., Sayed I.M., Abdelwahab S.F. (2022). Is Hepatitis E Virus a Neglected or Emerging Pathogen in Egypt?. Pathogens.

[35] Ibrahim E.H., Abdelwahab S.F., Nady S., Hashem M., Galal G., Sobhy M., Saleh A.S., Shata M.T. (2011). Prevalence of Anti-HEV IgM among Blood Donors in Egypt. Egypt. J. Immunol./Egypt. Assoc. Immunol..

[36] Tengan F.M., Figueiredo G.M., Nunes A.K.S., Manchiero C., Dantas B.P., Magri M.C., Prata T.V.G., Nascimento M., Mazza C.C., Abdala E. (2019). Seroprevalence of Hepatitis e in Adults in Brazil: A Systematic Review and Meta-Analysis. Infect. Dis. Poverty.

[37] dos Santos Weis-Torres S.M., de Oliveira França A., Granato C., Passarini A., Motta-Castro A.R.C. (2022). Seroprevalence of Hepatitis E Virus Infection among Volunteer Blood Donors in Central Brazil. Braz. J. Infect. Dis..

[38] Costa M.B., Gouvêa M.S.G., Chuffi S., Dellavia G.H., Ornel F., Von Diemen L., Kessler F., Pinho J.R.R., Álvares-da-Silva M.R. (2021). Seroprevalence of Hepatitis E Virus in Risk Populations and Blood Donors in a Referral Hospital in the South of Brazil. Sci. Rep..

[39] Moss da Silva C., Oliveira J.M., Mendoza-Sassi R.A., Figueiredo A.S., da Mota L.D., Nader M.M., Gardinali N.R., Kevorkian Y.B., Salvador S.B.S., Pinto M.A. (2019). Detection and Characterization of Hepatitis E Virus Genotype 3 in HIV-Infected Patients and Blood Donors from Southern Brazil. Int. J. Infect. Dis..

[40] Cunha G.G., Bezerra L.A., Silva Júnior J.V.J., Gonçales J.P., Montreuil A.C.B., Côelho M.R.C.D. (2022). Analysis of Seroprevalence and Risk Factors for Hepatitis E Virus (HEV) in Donation Candidates and Blood Donors in Northeast Brazil. Braz. J. Microbiol..

[41] Abdelaal M., Zawawi T.H., Al Sobhi E., Jeje O., Gilpin C., Kinsara A., Osoba A., Oni G.A. (1998). Epidemiology of Hepatitis E Virus in Male Blood Donors in Jeddah, Saudi Arabia. Ir. J. Med. Sci..

[42] Pittaras T., Valsami S., Mavrouli M., Kapsimali V., Tsakris A., Politou M. (2014). Seroprevalence of Hepatitis E Virus in Blood Donors in Greece. Vox Sang..

[43] Antonopoulou N., Schinas G., Kotsiri Z., Tsachouridou O., Protopapas K., Petrakis V., Petrakis E.C., Papageorgiou D., Tzimotoudis D., Metallidis S. (2024). Testing Hepatitis E Seroprevalence among HIV-Infected Patients in Greece: THE SHIP Study. Pathogens.

[44] Taherkhani R., Farshadpour F. (2016). Epidemiology of Hepatitis E Virus in Iran. World J. Gastroenterol..

[45] Ma Z., de Man R.A., Kamar N., Pan Q. (2022). Chronic Hepatitis E: Advancing Research and Patient Care. J. Hepatol..

[46] Izopet J., Lhomme S., Chapuy-Regaud S., Mansuy J.M., Kamar N., Abravanel F. (2017). HEV and Transfusion-Recipient Risk. Transfus. Clin. Biol..

[47] Damiris K., Meybodi M.A., Niazi M., Pyrsopoulos N. (2022). Hepatitis E in Immunocompromised Individuals. World J. Hepatol..

[48] Sayani F., Goldberg D., Slaven L., Russell J.E. (2015). Hepatitis E Infection in a Patient with Transfusion-Dependent β Thalassemia. Am. J. Hematol..

[49] Shah D., Nh B. (2018). Hepatitis E with Intravascular Hemolysis in Beta Thalassemia Trait: A Rare Association or Coincidence. J. Assoc. Physicians India.

[50] Koskinas J., Tampaki M., Dusheiko G., Porter J. (2023). Liver Disease. 2021 Guidelines: For the Management of Transfusion Dependent Thalassaemia (TDT).

[51] Boland F., Martinez A., Pomeroy L., O’Flaherty N. (2019). Blood Donor Screening for Hepatitis E Virus in the European Union. Transfus. Med. Hemotherapy.

[52] EASL (2019). Screening of Blood Donations for Hepatitis E Virus (HEV).

[53] Hoad V.C., Seed C.R., Fryk J.J., Harley R., Flower R.L.P., Hogema B.M., Kiely P., Faddy H.M. (2017). Hepatitis E Virus RNA in Australian Blood Donors: Prevalence and Risk Assessment. Vox Sang..

